# Validity and responsiveness of the Global Physical Activity Questionnaire (GPAQ) in assessing physical activity during pregnancy

**DOI:** 10.1371/journal.pone.0177996

**Published:** 2017-05-26

**Authors:** Estelle D. Watson, Lisa K. Micklesfield, Mireille N. M. van Poppel, Shane A. Norris, Matteo C. Sattler, Pavel Dietz

**Affiliations:** 1 Centre for Exercise Science and Sports Medicine, School of Therapeutic Sciences, Faculty of Health Sciences, University of Witwatersrand, Johannesburg, South Africa; 2 MRC/Wits Developmental Pathways for Health Research Unit, Department of Paediatrics, School of Clinical Medicine, Faculty of Health Sciences, University of Witwatersrand, Johannesburg, South Africa; 3 Institute of Sports Science, University of Graz, Graz, Austria; 4 Department of Public and Occupational Health, EMGO Institute for Health and Care Research, VU University Medical Centre, Amsterdam, the Netherlands; Universitat de les Illes Balears, SPAIN

## Abstract

The physiological and biomechanical changes that occur during pregnancy make accurate measurement of physical activity (PA) a challenge during this unique period. The Global Physical Activity Questionnaire (GPAQ) has been used extensively in low-to-middle income countries, but has never been validated in a pregnant population. In this longitudinal study, 95 pregnant women (mean age: 29.5±5.7 years; BMI: 26.9±5.0 kg/m^2^) completed the GPAQ and were asked to wear an accelerometer for 7 days at two time points during pregnancy (14–18 and 29–33 weeks gestation). There was a significant difference between accelerometry and GPAQ when measuring moderate-to-vigorous physical activity (MVPA) at 29–33 weeks gestation (16.6 vs 21.4 min/day; *p* = 0.02) as well as sedentary behaviour (SB) at both 14–18 weeks (457.0 vs 300 min/day; *p* < 0.01) and 29–33 weeks gestation (431.5 vs 300 min/day; *p* < 0.01). There was poor agreement between the GPAQ and accelerometry for both PA and SB at both time points (ICC: -0.05–0.08). Bland Altman plots indicated that the GPAQ overestimates PA by 14.8 min/day at 14–18 weeks and by 15.8 min/day at 29–33 weeks gestation. It underestimates SB by 127.5 min/day at 14–18 weeks and by 89.2 min/day at 29–33 weeks gestation. When compared to accelerometry, the GPAQ shows poor agreement and appears to overestimate PA and underestimate SB during pregnancy.

## Introduction

It is now generally accepted that regular physical activity (PA) has a fundamental role to play in positive health outcomes during pregnancy. Participating in 20–30 minutes of moderate intensity PA on most days of the week (approximately 150 minutes per week) [1], as recommended by the American College of Obstetricians and Gynecologists (ACOG) [2], provides various benefits such as reduced risk of excessive gestational weight gain, gestational diabetes mellitus [3,4] and preeclampsia [5]. In addition, women who are physically active during pregnancy benefit from increased cardiovascular fitness and muscular strength [6], as well as psychological benefits and improved mental health [7]. Although the evidence for the role of PA in birth outcomes is contradictory [8], some studies suggest that PA may have a protective effect on outcomes such as low birth weight, preterm birth and intrauterine growth restriction [9]. There is a general consensus that moderate intensity PA is safe to perform and does not place the fetus at any unnecessary risk [2,10].

The accurate measurement of PA is essential to monitor PA patterns, as well as to determine dose-response relationships and its associations with health outcomes in pregnancy [11]. Clarity on each of these aspects is critical for effective public health interventions, especially during pregnancy, but relies heavily on accurate measurement instruments. Self-reported questionnaires are simple, cost effective methods of assessing levels and patterns of PA in large samples [12, 13]. They are potentially useful to categorize participants as “active” or “inactive” within a population, and can provide useful information on activity domains such as occupational, transport or recreational PA [12]. However, the reliability and validity of questionnaires to measure PA have been called into question [13], and few appear to correlate well with the more objective measures of PA, such as accelerometry [14]. There are various questionnaires which have been used to assess PA during pregnancy, and the validity and reliability of many of these have been assessed [15, 16, 17]. Chasen-Taber et al. [17] developed the Pregnancy Physical Activity Questionnaire (PPAQ), which has been shown to be a moderately reliable measure of PA during this pregnancy. Correlations between the PPAQ and accelerometry cut points have varied between 0.08–0.58 for total PA, 0.20–0.49 for moderate intensity PA and 0.25–0.39 for vigorous intensity PA [18, 19]. In contrast, the International Physical Activity Questionnaire (IPAQ) has recently been found by Harrison et al. [15] to have a low correlation and poor absolute agreement with accelerometry during pregnancy. Similarly, Oostdam et al. [16] found very little association between accelerometry and the Activity Questionnaire for Adults and Adolescents (AQuAA), which appears to overestimate PA levels during pregnancy.

The Global Physical Activity Questionnaire (GPAQ) has been used extensively in low-to-middle-income countries (LMICs) [20], mostly due to its usefulness in capturing PA in various domains such as occupation, domestic tasks, walking for transport and recreation. Furthermore, a moderate agreement has been found with accelerometry for moderate-to-vigorous physical activity (MVPA, *r* = 0.04–0.48), but this was in non-pregnant populations [21, 22]. It has yet to be validated during pregnancy, despite being used to assess PA during the gestational period in some studies [23, 24]. The physiological and biomechanical changes that occur during pregnancy make accurate PA measurement a challenge [6], and a recent review by Poudevigne & O’Connor [7] found that much of the literature did not use validated measures of PA during pregnancy when assessing its relationship with health outcomes. Therefore, the aim of this study was to assess the use of the GPAQ to measure PA and SB at two time points during pregnancy (14–18 and 29–33 weeks gestation).

## Materials and methods

### Participants

This longitudinal, observational study included a subset of women recruited from a larger study, the Soweto First 1000 Days Cohort (S1000), based at the Medical Research Council (MRC)/Wits Developmental Pathways for Health Research Unit (DPHRU) which is located at the Chris Hani Baragwanath Hospital (CHBH) in Soweto, Johannesburg. Soweto is a large urban area in South Africa with mainly low income households. Women attending CHBH for antenatal care were included into this sub-study if they had healthy singleton pregnancies, and no contraindications to physical activity or exercise [2]. Data were collected at baseline (<14 weeks); 14–18 weeks gestation and 29–33 weeks gestation. Participants were provided with an information sheet and all participants signed a consent form for participation in the study. Approval to perform the study was provided by the Human Research Ethics Committee of the University of the Witwatersrand (Clearance number M130351).

### Measurements

Anthropometric measurements such as height (m) and weight (kg) were measured at baseline (<14 weeks) using a stadiometer and digital weighing scale (Seca, Hamburg, Germany). Body mass index (BMI; kg/m^2^) was calculated and classified according to the World Health Organization (WHO, 2000). Socioeconomic status was assessed by education, employment status, marital status and household inventory. The latter was based on the ownership of nine household commodities (electricity, radio, television, refrigerator, cell phone, personal computer, bicycle, motorcycle/scooter, car).

The GPAQ [20] was used to assess PA during work, travel and recreation at 14–18 weeks and 29–33 weeks gestation. Total time in MVPA was calculated according to the WHO STEPwise method and expressed as Metabolic Equivalent (METs) minutes per day (METmins/day). Furthermore, participants were classified as “active” if they accumulated ≥ 600 METmins/week or “inactive” if they did < 600 METmins/week (www.who.int/chp/steps/resources/GPAQ_Analysis_Guide). “Active” is the equivalent of reaching the recommended 150 minutes of moderate PA, 75 minutes of vigorous PA, per week or a combination of the two [1]. Sedentary behavior (SB, mins/day) was determined from the last question of the GPAQ which asks: “*How much time do you spend sitting/reclining on a typical day*?”

A triaxial accelerometer (ActiGraph GTX3+: 38x37x18mm, 27g; ActiGraph, Pensacola, FL) was used to assess PA in the form of activity counts at 14–18 weeks and 29–33 weeks gestation. The device was initialized to collect data at a sample rate of 30Hz and 15 second epochs. Participants were advised to wear the accelerometer on the right hip for at least seven consecutive days, and to remove the device when washing, bathing or sleeping. A minimum of 3 days and a maximum of 10 days of valid wear time (480 minutes of wear time per day), was required for inclusion into the analysis [25]. Analysis of the accelerometry data was done using ActiLife software (version 6) and Freedson cut points [26] were used to convert the accelerometer counts measured per minute into intensity bands, namely time of SB (< 100 counts/min), moderate intensity PA (1952–5724 counts/min) and vigorous intensity PA (> 5725 counts/min). Average daily time in MVPA (min/day) and SB (min/day) was calculated. These cut points have been commonly used in assessing validity of other questionnaires during pregnancy [19, 27]. Participants that performed 150 minutes of moderate or 75 minutes of vigorous PA per week were classified as “active” whilst women who did not reach these cut-offs were classified as “inactive.” Women were also classified into quartiles of MVPA based on the 25^th^, 50^th^, and 75^th^ percentile of MVPA min/day. Both accelerometery and GPAQ data was analyzed by one researcher (EDW).

### Validation process

In order to allow for comparisons with other validity studies, the Edinburgh Framework for validity and reliability [28], as well as the COSMIN checklist [29], were used where possible for the analysis. According to this, a broad range of different aspects of validity were considered for the present validation process, either by statistical analysis or theoretical approaches: content (face) validity (the degree to which the tool covers all the relevant aspects and dimensions of the construct), convergent validity (the degree of agreement with another tool that should assess the same parameter), relative validity (the ability of the tool to rank or categorize individuals within the parameter) and responsiveness (the ability of the tool to detect change over time) [28].

### Statistical analysis

Statistical analyses were performed using SPSS Data Analysis version 22.0 (IBM Corp., Armonk, NY) and MedCalc Version 16.2. (MedCalc Software, Ostend, Belgium). Descriptive statistics for all variables were calculated, including mean and standard deviation (SD) for normally distributed continuous variables, median and interquartile range (IQR) for non-normally distributed continuous variables, and frequencies and percentages for categorical variables. Histograms, Shapiro-Wilk tests and Q-Q-Plots indicated non-normality for 9 of 10 continuous PA variables, and therefore non-parametric tests were predominantly used for the analysis. Level of significance was defined as *p* < 0.05 for all analyses.

To allow for direct comparison between accelerometry and GPAQ, average min/day of MVPA or SB were calculated for both time points. Five outcome variables were considered for analysis, namely: SB (min/day) and MVPA (min/day) at both time points as well as change in MVPA (difference in MVPA min/day between 14–18 weeks and 29–33 weeks gestation), active/inactive classification of PA (based on MVPA min/day), and quartiles of PA (based on MVPA min/day) at both time points.

Responsiveness, convergent and relative validity, in particular the association between MVPA, SB and change in MVPA using GPAQ and accelerometry, was assessed by five different methods: Wilcoxon signed-rank test was used to compare median differences between the two tools; Intraclass correlation coefficient (ICC; two-way random effect model with absolute agreement definition) and Passing Bablok regression were used to assess the agreement of total PA and SB, and Kappa coefficients were used to assess the agreement of PA classifications (‘active/inactive and quartiles). The advantage of the non-parametric Passing Bablok regression is that it can test both proportional (variation of slope) and systematic differences (variation of intercept) separately and has no special assumptions concerning distribution and measurement errors [30]. The confidence interval (95%) of intercept and slope explains if the difference from 0 (intercept) and 1 (slope) is by chance [31]. Additionally, Bland-Altman plots were used to demonstrate the agreement between the PA and SB variables. Kappa coefficients were considered as slight agreement from 0–0.20, as fair agreement from 0.21–0.40, as moderate from 0.41–0.60, as substantial from 0.61–0.80 and as almost perfect from 0.81–1.00 [32]. ICC values greater than 0.9 indicate excellent agreement, whereas values from 0.75–0.9 indicate good agreement, values from 0.75–0.5 moderate and values below 0.5 indicate poor agreement [33].

To determine the minimal sample size required in order to detect an ICC of 0.5 between the two tools, a power analysis was performed with STATA 12.1 (Statacorp, College Station, TX, USA). To detect an ICC with a power of at least 80%, a sample size of *n* = 22 was required. To detect a moderate effect between the medians (*d* = 0.5) with a power of at least 80%, a sample size of *n* = 35 was required, as calculated with G*Power version 3 [33].

## Results

### Demographic characteristics

**Fig 1:**
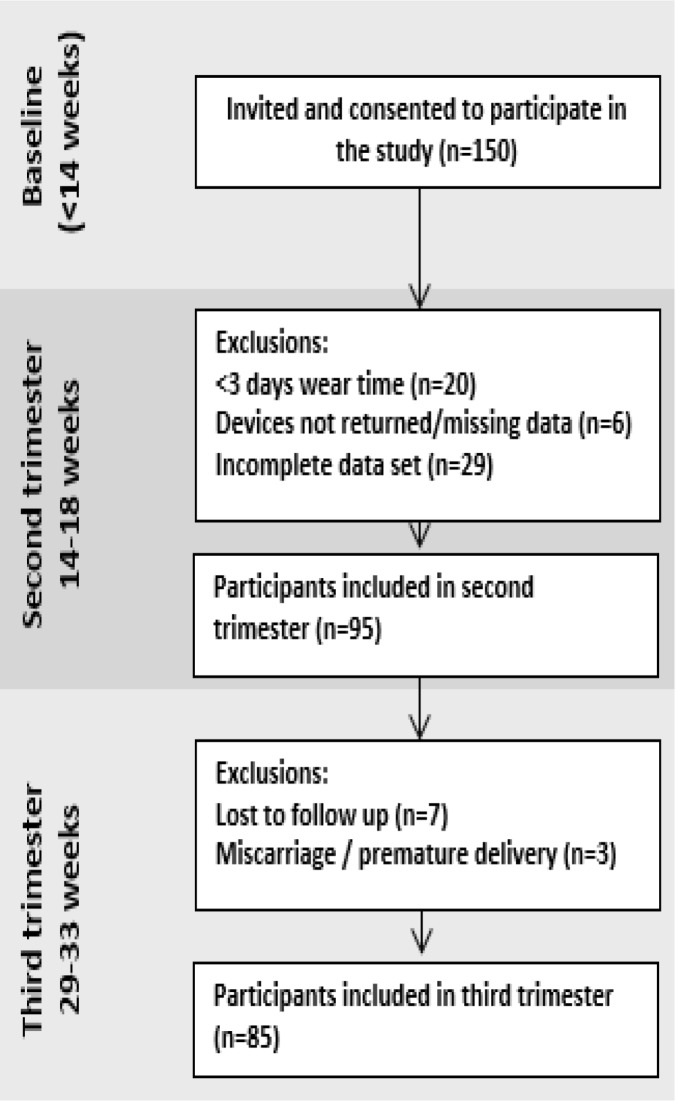
Participant flow and exclusions.

The participant flow and exclusions are presented in Fig 1. The demographic and anthropometric characteristics of the participants are presented in Table 1. Average wear time for the accelerometers was 6.8 days at both time points. The percentage of missing data/dropouts was low (0–6.3%) at 14–18 weeks gestation and low to medium (10.5%–25.3%) at 29–33 weeks gestation for all variables (Table 2). Comparison of missing and non-missing data showed no significant differences in age, BMI, education, household inventory, marital status, smoking, or occupation (data not shown).

**Table 1:** Anthropometric and demographic characteristics of the participants (n = 95).

		*Mean ± SD or %*
Age, *years*		29.5 ± 5.7
Height, *cm*		158.2 ± 6.2
Weight (<14 weeks), *kg*		67.4 ± 13.8
BMI (<14 weeks), *kg/m*^*2*^		26.9 ± 5.0
BMI Classification, *kg/m*^*2*^		
	Normal/underweight (≤25 kg/m^2^)	20.0%
	Overweight (25–30 kg/m^2^)	43.2%
	Obese (≥30 kg/m^2^)	35.8%
Weight gain (from <14 to 34–38 weeks), *kg*		8.7 ± 4.4
Marital status		
	Single/divorced	61.7%
	Married/cohabiting	38.3%
Children		
	None	31.9%
	1	39.4%
	2	21.3%
	3	7.4%
Household inventory, *# of items*		5.4 ± 1.2
Education level		
	Primary	5.3%
	Secondary	63.2%
	Tertiary	31.6%
Occupation		
	Manual work	18.9%
	Non-manual work	24.2%
	Unemployed/other	56.8%
Current smoker		
	No	90.5%
	Yes	9.5%

BMI = Body mass index; SD, standard deviation

**Table 2:** Physical activity variables measured using accelerometry and GPAQ.

Variable		14–18 weeks	29–33 weeks	*p-value*
		*Median (Interquartile range)*, *n*	*Median (Interquartile range)*, *n*	
*Accelerometry*				
	**MVPA**, *min/day*	26.2 (17.5–37.6), 92	16.6 (9.7–23.7), 74	< 0.01^a^
	**Sedentary behavior**, *min/day*	457.0 (380.4–546.5), 91	431.5 (312.0–500.1), 74	< 0.01^a^
*GPAQ*				
	**MVPA**, *METmin/day*	21.4 (10.7–42.9), 95	21.4 (7.1–41.4), 85	0.63^a^
	**Sedentary behavior**, *min/day*	300 (120–480), 89	300 (180–480), 83	0.61^a^
		*Percentage (n)*	*Percentage (n)*	***p-value***
*Accelerometry*				
	**Active classification**	66.3% (61)	35.1% (26)	< 0.01^b^
*GPAQ*				
	**Active classification**	50.5% (48)	50.6% (43)	0.99^b^

GPAQ, Global Physical Activity Questionnaire; MVPA, moderate-to-vigorous physical activity

^a^based on Wilcoxon Tests

^b^based on McNemar

### PA levels

Overall accelerometry and GPAQ PA and SB measurements for the participants are presented for both time points in Table 2. Median MVPA using accelerometry decreased significantly between the two timepoints (26.2 to 16.6 min/day; *p* < 0.01), whilst there was no significant difference in median MVPA as measured by the GPAQ. Similarly, median SB using accelerometry decreased significantly from 14–18 to 29–33 weeks gestation (457.0 to 431.5 min/day; *p* < 0.01), with no significant change in SB using the GPAQ. At 14–18 weeks, 66.3% of the participants were classified as active by accelerometry and this decreased to 35.1% at 29–33 weeks gestation (*p* < 0.01). No significant difference was found in the amount of participants that were classified as active by the GPAQ at 14–18 weeks (50.5%) compared to 29–33 weeks gestation (50.6%; *p* = 0.99).

### Convergent validity and responsiveness

**Fig 2:**
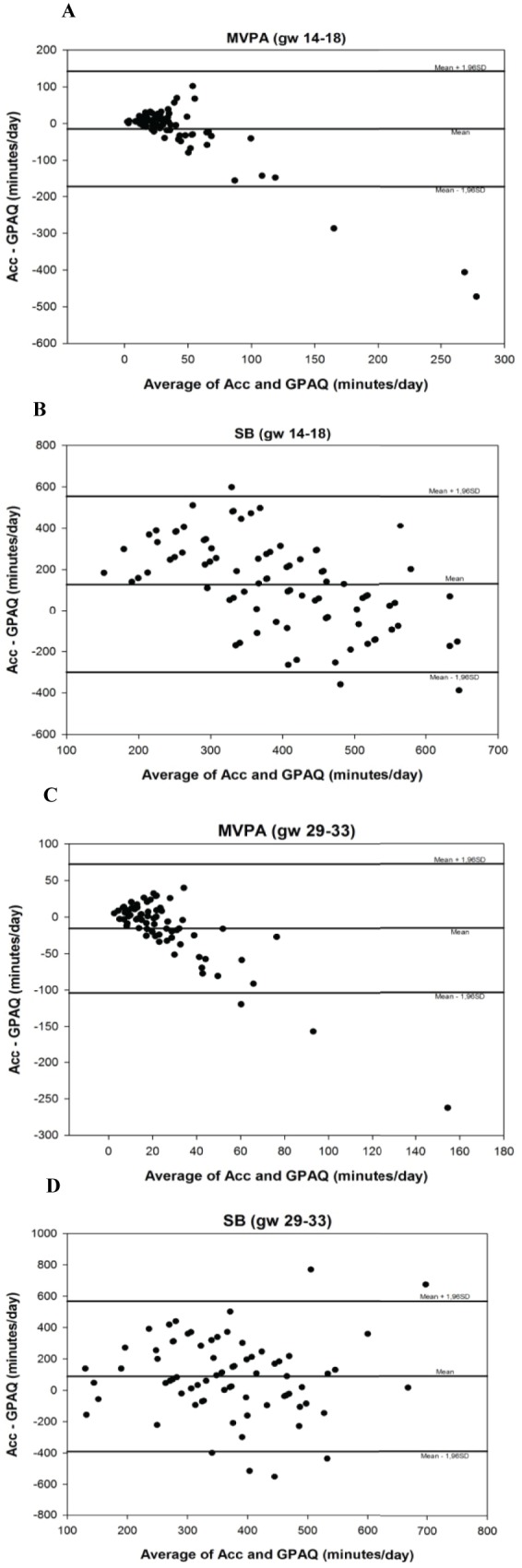
Bland-Altman plots demonstrating agreement between two PA measurements, Accelerometry (Acc) and Global Physical Activity Questionnaire (GPAQ), for MVPA (A and C), SB (B and D) and change in MVPA (E) at 14–18 wks, 29–33wks and change in MVPA, respectively.

Convergent validity results are presented in Table 3 and Fig 2. Wilcoxon signed-rank test showed significant differences between the medians of the two tools for MVPA (min/day) at 29–33 weeks gestation (*p* = 0.02), and SB (min/day) at both time points (*p* < 0.01). No significant differences were found between the two tools for MVPA (min/day) at 14–18 weeks gestation (*p* = 0.81) or change in MVPA (*p* = 0.14). The ICC ranged from -0.05 to 0.08 for all PA and SB variables (*p* > 0.05), indicating a poor agreement between the two tools. Likewise, results from the Passing Bablok regression indicated poor agreement for all PA and SB variables, with the intercept ranging from -1255.5 to 103.9, and the slope from 1.5 to 7.8, displaying both proportional and systematic difference between accelerometry and GPAQ. SB (min/day) at 29–33 weeks gestation was the only variable with a slope that was close to 1 (1.5; 95%CI: 0.8–2.5). In addition, MVPA (min/day) at 29–33 weeks gestation and SB (min/day) at 14–18 weeks gestation showed significant deviations from linearity (*p* = 0.01 and *p* < 0.01 respectively), indicating a non-linear relationship between the two tools.

**Table 3:** Main results of convergent and relative validity for GPAQ, compared to Accelerometer, measuring PA during pregnancy.

	Wilcoxon	ICC^a^	Passing Bablok Regression			Kappa (*κ*)
	*Z*, *p-value*	*ICC [95% CI]*, *p-value*.	*Intercept [95% CI]*.	*Slope [95% CI]*.	*Linearity*	*κ (SE)*, *p-value*.
MVPA min/day (14–18wks)	-0.24, 0.81	0.08 [-0.12–0.28], 0.21	-33.84 [-78.49 –-15.08]	2.36 [1.61–4.05]	*p* = 0.13	
MVPA min/day (29–33wks)	-2.44, 0.02*	0.01 [-0.19–0.22], 0.47	-69.92 [-238.84 –-18.97]	5.55 [2.62–16.95]	*p* = 0.01*	
SB min/day (14–18wks)	-4.67, < 0.01*	0.05 [-0.11–0.22], 0.26	-1255.45 [-2355.30 –-694.92]	3.46 [2.18–5.84]	*p* < 0.01*	
SB min/day (29–33wks)	-3.16, < 0.01*	-0.05 [-0.25–0.16], 0.69	-255.08 [-672.24 –-13.25]	1.45 [0.81–2.45]	*p* = 0.87	
Change in MVPA min/day	-1.50, 0.14	0.04 [-0.20–0.27], 0.36	103.92 [39.75–233.90]	7.83 [3.80–19.62]	*p* = 0.10	
Active/Inactive (14–18wks)	-	-	-	-	-	0.11 (0.10), 0.27
Active/Inactive (29–33wks)	-	-	-	-	-	-0.02 (0.11), 0.86
Quartiles of PA (14–18 wks)	-	-	-	-	-	0.09 (0.07), 0.15
Quartiles of PA (29–33 wks)	-	-	-	-	-	-0.03 (0.07), 0.70

^a^ Intraclass correlation coefficient: one-way Random Effect Model with absolute agreement definition.

* p < 0.05.

Bland Altman plots (Fig 2) revealed mean differences between accelerometry and GPAQ for MVPA at 14–18 weeks gestation (-14.8 min/day; 95%CI: -172.0–142.4), MVPA at 29–33 weeks gestation (-15.8 min/day; 95%CI: -103.9–72.4). Therefore, it appears that the GPAQ on average overestimates MVPA by 14.8 min/day in the second trimester of pregnancy and by 15.8 min/day in the third trimester. The plots indicate that this overestimation appears to increase after around 20–25 min/day of MVPA.

For SB the mean differences between accelerometry and GPAQ was 127.5 min/day (95%CI: -299.2–554.2) at 14–18 weeks gestation and 89.2 min/day (95%CI:-390.7–569.2) at 29–33 weeks gestation, indicating that the GPAQ underestimates the amount of SB by an average of 127.5 min/day in the second trimester and 89.2 min/day in the third trimester. The mean difference in change in MVPA between the two tools was 2.2 min/day (95%CI: -200.6–205.1), and this difference appeared to increase with an increasing change in MVPA min/day between the second and third trimester. Moreover, the plots indicate wide limits of agreements for both MVPA and SB at all time points.

### Relative validity

Comparison of the active/inactive classification based on MVPA showed non-significant Kappa coefficients of -0.02 (*p* = 0.86) and 0.11 (*p* = 0.27), thus, indicating a poor agreement between accelerometry and GPAQ. Furthermore, the same poor agreement was found between the two tools when classifying PA according to quartiles, at both time points (Table 3). The area under the curve (AUC) was 0.56 (95%CI: 0.44–0.69) for active/inactive classification at 14–18 weeks gestation (sensitivity: 58.1%, specificity: 54.1%) and 0.49 (95%CI: 0.35–0.63) for active/inactive classification at 29–33 weeks gestation (sensitivity: 47.9%, specificity: 50.0%).

## Discussion

The current study is the first of its kind to report different measures of validity for the GPAQ in comparison to accelerometry in measuring PA and SB during pregnancy. Accurately measuring PA and SB is a multi-faceted concern and describing issues of validity is especially complex. In a recent paper by Kelly et al. [28], the approach to assessing and describing validity of PA measurements has been brought into question, in order to ensure clarity, accuracy and comparability between different validation studies of PA. In the current study, convergent and relative validity was assessed using five different methods, and the findings of the current study indicate a poor validity of GPAQ when compared to accelerometry for measuring PA and SB as well as a poor responsiveness for PA. For instance, on average the GPAQ overestimated MVPA by 14.8min/day in the second trimester and 15.8min/day in the third trimester. In addition, the GPAQ appears to underestimate SB more in the second than the third trimester. Consequently, the GPAQ may not be the most suitable questionnaire for measuring PA in pregnancy in this population, and should be used with caution.

### Content and face validity

Content validity in the current study indicates the extent to which the GPAQ covers all relevant aspects or domains of PA and SB in pregnancy [28]. Measuring and describing PA is a complex issue, which needs to encompass different domains (e.g. travel, occupation, leisure time, housework and gardening or caregiving), dimensions (duration, frequency, intensity, type) and correlates (where, when, who, why) [28]. The validity of accelerometers to capture different types of activity such as cycling or swimming, and to differentiate between sitting at work and sitting watching television, may be questionable [28], and therefore self-report questionnaires may provide insight into these behavioral complexities of PA. Indeed, the GPAQ provides useful information about different domains (e.g. work, transport, leisure) of PA as well as information about duration (hours and minutes per day), and frequency (days per week). However, it may not provide domain-specific information relevant to pregnancy that is available in other questionnaires [17], for example care-giving, preparing meals and feeding children. The latter is especially important for pregnant women, who already have children at home, since PA levels have been shown to differ according to parity [34]. Therefore, the lack of pregnancy-specific dimensions may lead to an underestimation of PA levels when using the GPAQ during pregnancy. It appears from previous research [17, 27] that questionnaires that include specific domains relevant during pregnancy, for example childcare, could provide better content and construct validity than the GPAQ. Content and face validity is not only a matter of the chosen measurement instrument (e.g. self-report, accelerometry, doubly labeled water) but also a matter of the study purpose. Measuring PA for different study purposes (e.g. total PA, PA in different domains, PA during different stages of the life course) means that different measurement instruments should be considered. Clearly, PA in pregnancy represents a distinct life stage, probably with the necessity of specific accelerometry techniques and questionnaires.

### Convergent validity and responsiveness

According to a review of 12 studies by Evenson et al. [27], questionnaires demonstrated a poor to moderate agreement when compared to objective measures of PA during pregnancy. Previous studies have found similar correlations of 0.08 [17] and 0.14 [16] when assessing total PA using accelerometers analysed with Freedson cut points, and questionnaires. Likewise, the current study showed poor agreement between GPAQ and accelerometry measures for MVPA when assessed with different statistical and graphical methods, which is in line with results from previous findings [28, 35].

One explanation is that the GPAQ is designed for assessment of PA levels in the general population, whilst some previous validation studies have used pregnancy-specific questionnaires such as the PPAQ or Pregnancy Infection and Nutrition 3 (PIN3) recall questionnaires [27]. As discussed previously, since the GPAQ omits some pregnancy-related domains of PA, such as feeding, carrying and playing with children, this may contribute towards the low agreement found in the current study.

In addition, within the development of the PPAQ, Chasen-Taber et al. [17] found higher correlations when using Hendelman et al. (0.43) and Swartz et al. (0.32) cut points, when compared to those of Freedson et al. (0.08), indicating the importance of data processing when using accelerometry to assess validity of self-reported PA [17]. Therefore, although Freedson cut points are widely used in validation studies during pregnancy [27], they may not be appropriate, given the physiological and biomechanical changes that occur. Since no pregnancy-specific cut points currently exist, further research is needed before the most accurate cut points for this period can be decided upon.

Furthermore, little is known about the validity and reliability of accelerometry during pregnancy [35, 36]. Although accelerometry has been validated against the claimed “gold standard” of doubly labeled water in non-pregnant populations, it is well known that the energy costs of pregnancy differ significantly to that of the general population [37], and increase with gestation [38] thereby potentially affecting the accuracy of accelerometry for measuring energy expenditure.

Pregnancy appears to be a vulnerable time for SB, with both measures indicating that women spend between 5–7.6 hours per day in sedentary behaviours. This is similar to US data, where Evenson et al. [39] found that 15.3% of pregnant women spend 5 hours per day watching television. Very few validation studies have assessed SB during pregnancy, and those that have reported correlations of 0.23 to 0.78 between self-report and objective measures [27]. The current study showed poor agreement between self-reported SB when compared to accelerometer-measured SB at both time points (ICC: 0.05 and -0.05). Moreover, the GPAQ appeared to underestimate SB by 127.5 min/day and 89.2 min/day in the second and third trimesters respectively.

Similar to previous findings [7, 40], this study found that accelerometer-measured PA levels declined during pregnancy, indicating that women become more inactive with gestation. In the present study, the agreement to these changes in PA was low between accelerometry and GPAQ, indicating a poor responsiveness for PA. Declining levels of PA during pregnancy have been explained by increasing belly size, discomfort and fatigue [7]. Indeed, these physiological changes may affect the agreement between self-report and accelerometry, since women may perceive their light activities to be at a moderate-to-vigorous intensity, leading to the overestimation of MVPA. On the other hand, the physiological changes may mean that women are indeed working at a higher intensity, but the accelerometry cut points are not sensitive enough to detect these increases in energy expenditure with the same acceleration. Furthermore, the GPAQ uses MET values to calculate intensity during pregnancy, however these are based on energy expenditure in the non-pregnant population, and may not appropriately account for the physiological and cardiovascular changes that occur during pregnancy [6].

### Relative validity

The prevalence of pregnant women meeting the recommended PA guidelines was higher (35–66%) in this study than previously reported data (22–44%) [39, 41], using both the GPAQ and accelerometer measures of PA. From a public health perspective, individual PA levels are often used to classify populations into “active” and “inactive” in order to highlight the need for an intervention [12]. In this study, GPAQ and accelerometry displayed poor agreement within this active/inactive classification. In addition, it also displayed poor agreement in detecting quartiles of PA. These comparisons indicate poor relative validity of the GPAQ for both trimesters.

### Limitations and strengths of the study

First, while keeping in mind the association between reliability and validity, this study did not assess the reliability of the GPAQ and accelerometer in measuring PA during pregnancy, which may in turn affect the validity of both instruments. Furthermore, as Kelly et al. [28] have described, there is much complexity in the dimensions assessed between various PA measurement tools. Therefore, direct comparison between two different PA assessment tools is not possible, and the conclusion of validity of this study is based upon comparing the inferred outcomes of each tool, rather than the tool itself. Secondly, South Africa, and Soweto in particular, is a culturally diverse area, with eleven official languages, and although English is the most widely spoken language, it may not have been the home language for many of the participants, thereby affecting its internal validity in the current study. Lastly, since the participants in the current study were all black, African women from a low to middle socioeconomic background, it does not present a sufficiently diverse population to provide external validity, and therefore generalizability, to other population groups.

On the other hand, previous validation studies have mostly originated from high-income countries such as the Netherlands, Norway and the United States, and this is the first study of its kind to assess the GPAQ as a measurement tool for PA and SB during pregnancy in an African, LMIC setting. In addition, the sample size allowed for the analysis of validity with sufficient degree of accuracy, and the longitudinal nature of the study allowed for assessment of responsiveness, which has not been commonly done during pregnancy. This study specifically assessed the many different aspects of validity, using the Edinburgh Framework [28] and COSMIN [29, 42], in order to clarify the approach of validity assessment and allow for future comparisons. This was achieved by using various statistical and graphical methods and combining these with theoretical approaches to consider a holistic approach to validation.

## Conclusion

Pregnancy is a unique life stage in which to measure free-living PA. In this longitudinal study of pregnant black South African women, the GPAQ has poor agreement with accelerometry when measuring PA, SB or change in PA. Other questionnaires, such as the PPAQ may provide more content/face validity, but may not be as applicable in a LMIC setting. Development of a pregnancy-specific questionnaire for women in LMIC settings may be needed. Further research is warranted to confirm the accuracy of accelerometry and valid cut points during pregnancy. In the meantime, researchers should apply the use of GPAQ during pregnancy with some caution.

## Supporting information

S1 Fig Study data set. (PDF)
