# Supplementary material for: Validity and responsiveness of the Global Physical Activity Questionnaire (GPAQ) in assessing physical activity during pregnancy
Source: PLoS One. 2017 May 26;12(5):e0177996. doi: 10.1371/journal.pone.0177996 (PMC5446115; doi:10.1371/journal.pone.0177996)
Supplement: S1 Fig — (PDF) [file pone.0177996.s001.pdf]

Patient ID	Age	Height <14 weeks(cm)	Weight at <14 weeks (kg)	Weight at 14-18 weeks (kg)	Weight at 19-23 weeks (kg)	Weight at 24-28 weeks (kg)	Weight at 29-33 weeks (kg)	Weight at 34-38 weeks (kg)	Weight gain 34-38 weeks (kg)	BMI <14 weeks	BMI Classification	Marital Status	Level of Education	Emplyment Status
GPAQ001	21.00	148.50	65.90	69.00	74.50	80.40	84.40			29.88	Overweight	Married/cohab	Secondary	Nonmanual
GPAQ002	23.00	164.30	79.80	77.70	79.10	83.00	84.50	87.80	8.00	29.56	Overweight	Single/divorced	Secondary	Nonmanual
GPAQ003	25.00	153.40	53.40	56.40	56.70	55.50	56.10	60.00	6.60	22.69	Normal	Single/divorced	Secondary	Manual work
GPAQ004	31.00	160.70	65.90	67.30	70.60	70.80	72.10	75.90	10.00	25.52	Overweight	Single/divorced	Secondary	Manual work
GPAQ005	27.00	142.60	56.10	58.70	63.10	64.30	67.30	69.40	13.30	27.59	Overweight	Married/cohab	Secondary	Nonmanual
GPAQ006	37.00	159.10	85.10	83.90	84.00	82.30	86.60			33.62	Obese	Married/cohab	Secondary	Unemployed/o
GPAQ007	29.00	155.80	63.80	65.10	66.30	68.30	70.40	79.30	15.50	26.28	Overweight	Single/divorced	Proffessional/t	Nonmanual
GPAQ008	32.00	163.30	64.00	67.20	72.40	76.00	81.90			24.00	Normal	Single/divorced	Secondary	Manual work
GPAQ009	37.00	165.70	73.50	76.60	76.90	78.10	80.40			26.77	Overweight	Married/cohab	Secondary	Nonmanual
GPAQ010	24.00	155.10	67.90	70.30	72.40	76.20	77.70	82.90	15.00	28.23	Overweight	Single/divorced	Proffessional/t	Nonmanual
GPAQ011	35.00	165.10	93.30	95.10	98.70	98.70	101.60	101.50	8.20	34.23	Obese	Single/divorced	Proffessional/t	Manual work
GPAQ012	41.00	156.70	58.80	60.00	62.20	63.50	64.90	66.90	8.10	23.95	Normal	Married/cohab	Secondary	Unemployed/o
GPAQ013	24.00	167.10	56.50	57.90	60.50	62.60	65.10			20.23	Normal	Married/cohab	Proffessional/t	Nonmanual
GPAQ014	29.00	166.70	78.50	81.40	85.80	89.50	92.30	94.50	16.00	28.25	Overweight	Married/cohab	Proffessional/t	Unemployed/o
GPAQ015	34.00	154.30	68.80	68.80	71.20	71.60	74.70	74.70	5.90	28.90	Overweight	Married/cohab	Secondary	Unemployed/o
GPAQ016	37.00	161.00	64.10	61.70	61.80	62.00	64.30	65.20	1.10	24.73	Normal	Single/divorced	Secondary	Unemployed/o
GPAQ017	31.00	152.00	59.00	59.50	65.10	67.50	70.80	75.10	16.10	25.54	Overweight	Single/divorced	Secondary	Nonmanual
GPAQ018	30.00	159.00	71.00	72.10	74.70	77.20	78.90	78.90	7.90	28.08	Overweight	Married/cohab	Secondary	Unemployed/o
GPAQ019	32.00	163.00	49.60	48.80	51.80	53.70	57.00	59.90	10.30	18.67	Normal	Single/divorced	Secondary	Unemployed/o
GPAQ020	39.00	166.60	86.40	87.30	89.10	89.60	91.60	92.10	5.70	31.13	Obese	Single/divorced	Proffessional/t	Manual work
GPAQ021	33.00	162.30	62.50	62.30	65.70	70.20	72.60	72.60	10.10	23.73	Normal	Married/cohab	Secondary	Unemployed/o
GPAQ022	34.00	166.00	54.00	56.10	60.00	62.40	66.20	68.50	14.50	19.60	Normal	Married/cohab	Secondary	Nonmanual
GPAQ023	27.00	154.40	45.50	47.60	49.80	52.80	53.30	53.30	7.80	19.09	Normal	Single/divorced	Proffessional/t	Nonmanual
GPAQ024	21.00	156.80	46.80	46.70	48.00	49.20	49.90	54.00	7.20	19.04	Normal	Single/divorced	Secondary	Unemployed/o
GPAQ025	27.00	148.90	60.00	61.90	64.90	64.70	64.30	64.30	4.30	27.06	Overweight	Married/cohab	Secondary	Unemployed/o
GPAQ026	29.00	164.90	94.10	96.10						34.61	Obese	Married/cohab	Secondary	Nonmanual
GPAQ027	36.00	170.00	73.70	75.60	80.30	88.60	91.30	91.30	17.60	25.50	Overweight	Single/divorced	Secondary	Nonmanual
GPAQ028	32.00	151.50	61.70	62.80	65.00	65.80	66.40	67.20	5.50	26.88	Overweight	Married/cohab	Proffessional/t	Unemployed/o
GPAQ029	30.00	164.80	49.50	51.70	53.80	59.90	60.40	64.50	15.00	18.23	Underweight	Single/divorced	Secondary	Unemployed/o
GPAQ030	34.00	148.90	70.50	73.40	75.60	77.20	79.60	81.20	10.70	31.80	Obese	Married/cohab	Proffessional/t	Unemployed/o
GPAQ031	24.00	156.30	63.10	64.60	66.40	68.90	72.70	76.00	12.90	25.83	Overweight	Single/divorced	Secondary	Unemployed/o
GPAQ032	37.00	158.20	74.70	72.20	80.20	83.10	86.50	90.50	15.80	29.85	Overweight	Married/cohab	Secondary	Unemployed/o
GPAQ033	35.00	161.80	60.10	61.50	65.90	67.20	70.10	73.10	13.00	22.96	Normal	Single/divorced	Secondary	Nonmanual
GPAQ034	28.00	154.90	55.80	56.50	60.40	62.50	65.30	69.30	13.50	23.26	Normal	Married/cohab	Primary	Unemployed/o
GPAQ035	41.00	150.60	55.20	55.10	57.60			60.70	5.50	24.34	Normal	Single/divorced	Secondary	Unemployed/o
GPAQ036	20.00	160.80	69.60	70.50	72.30	74.90	78.60	82.00	12.40	26.92	Overweight	Married/cohab	Secondary	Unemployed/o
GPAQ037	28.00	164.00	79.40	79.80	82.60	85.60	85.60	88.50	9.10	29.52	Overweight	Single/divorced	Proffessional/t	Manual work
GPAQ038	31.00	155.10	71.80	73.50						29.85	Overweight	Married/cohab	Secondary	Unemployed/o

GPAQ039	28.00	160.10	72.70	72.20	72.80	72.40	72.40	74.00	1.30	28.36	Overweight	Married/cohab	Secondary	Unemployed/o
GPAQ040	35.00	160.70	66.20	68.50	68.50	69.10	71.70	71.70	5.50	25.63	Overweight	Single/divorced	Proffessional/t	Nonmanual
GPAQ041	41.00	166.70	82.70	81.40	82.60	84.10	82.90	81.30	-1.40	29.76	Overweight	Single/divorced	Secondary	Manual work
GPAQ042	27.00	161.60	78.80	80.60	80.70	84.30		84.30	5.50	30.17	Obese	Single/divorced	Secondary	Manual work
GPAQ043	24.00	149.90	68.90	69.50	70.60	71.90	72.40	72.40	3.50	30.66	Obese		Secondary	Unemployed/o
GPAQ044	33.00	162.30	72.80	75.00	75.60	77.20		77.20	4.40	27.64	Overweight	Single/divorced	Secondary	Unemployed/o
GPAQ045	22.00	162.30	68.00	68.40	72.00	74.40	86.10	77.50	9.50	25.81	Overweight	Single/divorced	Proffessional/t	Unemployed/o
GPAQ046	36.00	155.10	51.20	53.60	56.20	56.10	57.80	61.00	9.80	21.28	Normal	Single/divorced	Secondary	Unemployed/o
GPAQ047	21.00	158.70	53.40	54.90	57.50	61.80	65.20	70.20	16.80	21.20	Normal	Single/divorced	Secondary	Unemployed/o
GPAQ048	29.00	149.00	63.80	65.30	68.20	71.80	74.90	75.20	11.40	28.74	Overweight	Married/cohab	Proffessional/t	Nonmanual
GPAQ049	24.00	154.30	71.30	72.40	74.50	75.50	79.50	83.60	12.30	29.95	Overweight	Single/divorced	Secondary	Unemployed/o
GPAQ050	28.00	157.60	73.00	73.20	74.40	77.60	79.30	82.00	9.00	29.39	Overweight	Married/cohab	Secondary	Unemployed/o
GPAQ051	34.00	157.80	59.70	60.30	62.00	63.00	65.30	66.20	6.50	23.98	Normal	Single/divorced	Secondary	Unemployed/o
GPAQ052	26.00	144.60	67.20	67.30	68.60	68.70	71.70	70.20	3.00	32.14	Obese	Single/divorced	Proffessional/t	Manual work
GPAQ053	24.00	158.00	74.40	77.50	82.10	84.40	87.20	91.10	16.70	29.80	Overweight	Single/divorced	Secondary	Unemployed/o
GPAQ054	30.00	158.00	45.70	55.50	49.40	53.00	55.30	57.00	11.30	18.31	Underweight	Single/divorced	Secondary	Manual work
GPAQ055	31.00	160.60	55.20	57.60	60.50	63.40	64.00	64.00	8.80	21.40	Normal	Single/divorced	Secondary	Unemployed/o
GPAQ056	27.00	169.50	82.10	82.40	83.60	87.30	91.60	91.60	9.50	28.58	Overweight	Married/cohab	Proffessional/t	Unemployed/o
GPAQ057	37.00	152.30	64.20	61.40	67.90					27.68	Overweight	Single/divorced	Secondary	Unemployed/o
GPAQ058	34.00	152.80	70.90	73.50	74.80	76.10	75.80	78.00	7.10	30.37	Obese	Married/cohab	Secondary	Unemployed/o
GPAQ059	27.00	161.90	50.90	52.30	53.50	56.10	58.30	58.30	7.40	19.42	Normal	Single/divorced	Secondary	Unemployed/o
GPAQ060	27.00	165.40	87.50	86.70	88.40	91.00	92.80	94.00	6.50	31.98	Obese	Married/cohab	Proffessional/t	Unemployed/o
GPAQ061	43.00	156.70	68.20	70.90	70.10	71.30	72.30	73.80	5.60	27.77	Overweight	Single/divorced	Proffessional/t	Unemployed/o
GPAQ062	28.00	161.10	69.20	70.00	71.80	73.40	74.10	75.40	6.20	26.66	Overweight	Single/divorced	Secondary	Manual work
GPAQ063	38.00	154.00	70.00	72.90	72.50	71.20	72.80	72.80	2.80	29.52	Overweight	Single/divorced	Primary	Unemployed/o
GPAQ064	30.00	151.10	65.60	66.40	67.90	67.90		67.90	2.30	28.73	Overweight	Single/divorced	Secondary	Unemployed/o
GPAQ065	24.00	165.90	61.10	62.00	63.20	66.80		71.50	10.40	22.20	Normal	Single/divorced	Proffessional/t	Unemployed/o
GPAQ066	39.00	155.80	78.70	74.60	77.00	78.10	77.80	77.80	-0.90	32.42	Obese	Single/divorced	Primary	Unemployed/o
GPAQ067	26.00	147.00	53.60	57.40	60.10	63.00	66.30	66.30	12.70	24.80	Normal	Single/divorced	Secondary	Manual work
GPAQ068	32.00	166.60	64.70	64.70						23.31	Normal	Married/cohab	Secondary	Manual work
GPAQ069	32.00	163.10	72.00	73.10	75.30	76.90	78.00	80.10	8.10	27.07	Overweight	Single/divorced	Secondary	Unemployed/o
GPAQ070	23.00	160.80	57.70	61.70	64.60	68.50	69.60	70.90	13.20	22.32	Normal	Single/divorced	Secondary	Nonmanual
GPAQ071	30.00	158.20	74.50	76.60	77.70	79.70	81.10	83.50	9.00	29.77	Overweight	Married/cohab	Secondary	Manual work
GPAQ072	31.00	167.40	89.60	94.00	93.10	94.70	100.40	100.90	11.30	31.97	Obese	Married/cohab	Proffessional/t	Unemployed/o
GPAQ073	31.00	153.50	44.90	46.00	45.90	47.70	48.70	49.10	4.20	19.06	Normal	Single/divorced	Primary	Unemployed/o
GPAQ074	20.00	156.20	60.20	61.90	63.80	63.40	66.00			24.67	Normal	Married/cohab	Proffessional/t	Nonmanual
GPAQ075	27.00	158.20	75.70	76.50	77.30	79.80	84.10	85.70		30.25	Obese	Married/cohab	Proffessional/t	Unemployed/o
GPAQ076	32.00	153.40	53.30	58.20	63.50	63.50	63.30	66.10		22.65	Normal	Single/divorced	Secondary	Manual work
GPAQ077	27.00	143.90	55.40	56.40	57.30	58.40	59.40	60.60		26.75	Overweight	Single/divorced	Primary	Unemployed/o
GPAQ078	29.00	156.30	59.10	60.80	63.30	64.40	67.80			24.19	Normal	Married/cohab	Secondary	Unemployed/o
GPAQ079	24.00	156.80	50.80	53.40	55.60	56.30	59.50	59.50	8.70	20.66	Normal	Single/divorced	Proffessional/t	Nonmanual
GPAQ080	24.00	157.40	52.10	53.00	56.50	56.90	58.80	56.90	4.80	21.03	Normal	Single/divorced	Secondary	Unemployed/o

GPAQ081	24.00	146.10	47.10	47.60	49.50	50.60	52.50	56.30	9.20	22.07	Normal	Single/divorced	Secondary	Unemployed/o
GPAQ082	23.00	159.80	48.00	48.00	49.70	50.00		54.40	6.40	18.80	Normal	Single/divorced	Proffessional/te	Nonmanual
GPAQ083	24.00	165.20	71.60	73.60	74.30	74.60	75.00	75.00	3.40	26.24	Overweight	Single/divorced	Proffessional/te	Nonmanual
GPAQ084	22.00	157.00	87.30	89.00	90.90	92.90	95.10	95.10	7.80	35.42	Obese	Married/cohab	Proffessional/te	Unemployed/o
GPAQ085	22.00	155.70	98.80	102.30	105.70	107.30	109.20	111.10	12.30	40.75	Obese	Single/divorced	Secondary	Manual work
GPAQ086	21.00	157.80	71.90	73.20	77.10	80.60	85.50	87.40	15.50	28.87	Overweight	Single/divorced	Proffessional/te	Nonmanual
GPAQ087	20.00	165.70	55.60	60.40	61.30	62.50	67.00			20.25	Normal	Single/divorced	Proffessional/te	Manual work
GPAQ088	22.00	163.00	82.40	83.20	84.30	86.90	88.20	90.60	8.20	31.01	Obese	Single/divorced	Proffessional/te	Nonmanual
GPAQ089	29.00	147.60	56.40	53.20	55.50	57.60	59.80	59.80	3.40	25.89	Overweight	Married/cohab	Proffessional/te	Unemployed/o
GPAQ090	34.00	166.00	70.90	73.10	75.00	76.30	79.30	79.30	8.40	25.73	Overweight	Married/cohab	Proffessional/te	Unemployed/o
GPAQ091	36.00	151.90	54.90	56.10	57.40	59.80	58.30	58.30	3.40	23.79	Normal	Single/divorced	Secondary	Unemployed/o
GPAQ092	30.00	155.00	86.60	88.30	90.80	93.00	96.40	96.40	9.80	36.05	Obese	Single/divorced	Secondary	Nonmanual
GPAQ093	37.00	157.10	92.70	94.60	96.70	96.20	96.50			37.56	Obese	Married/cohab	Secondary	Manual work
GPAQ094	35.00	160.70	101.70	101.10	104.20	101.90	104.90			39.38	Obese	Single/divorced	Secondary	Unemployed/o
GPAQ095	24.00	166.90	114.20	111.90	114.60	116.10				41.00	Obese	Married/cohab	Secondary	Unemployed/o

Household inventory (/9)	Number of Children	V2_P16_Total	V2_PtotalMIN/	Quartiles of PA_GPAQ	V2_Ptotal_MET	V2_Ptotalday_	V2_GPAQ Mee	Sedentary	Light	Total MVPA	V2_Acceleromo	Accelerometer	Quartiles of ac
3.00	0.00	30.00	38.57	3.00	1080.00	154.29	Active	2632.5	836	275.5	1.00	34.438	3.00
5.00	0.00	480.00	10.71	2.00	300.00	42.86	Inactive	3796.5	1110.75	176.75	1.00	25.25	2.00
1.00	1.00	240.00	25.71	3.00	720.00	102.86	Active	2805.75	899.75	183.5	1.00	22.938	2.00
6.00	0.00	720.00	12.86	2.00	360.00	51.43	Inactive	4372.25	1626	359.75	1.00	44.969	4.00
7.00	1.00	600.00	25.71	3.00	720.00	102.86	Active	4666.75	1038.5	297.75	1.00	42.536	4.00
5.00	2.00	420.00	10.71	2.00	300.00	42.86	Inactive	3362.25	2324.75	474	1.00	67.714	4.00
7.00	0.00		7.14	1.00	200.00	28.57	Inactive	2976.75	977.75	288.5	1.00	36.063	3.00
6.00	1.00	840.00	5.71	1.00	160.00	22.86	Inactive	3617.5	1663.25	94.25	0.00	11.781	1.00
6.00	0.00	120.00	28.57	3.00	800.00	114.29	Active	4144.25	1059.25	163.5	1.00	23.357	2.00
7.00	1.00	30.00	68.57	4.00	1920.00	274.29	Active	1996.25	508	100.75	0.00	20.15	2.00
7.00	2.00	480.00	27.14	3.00	760.00	108.57	Active	4749.75	1396.75	140.5	0.00	20.071	2.00
5.00	3.00	480.00	42.86	4.00	1200.00	171.43	Active	3860.25	990.5	169.25	1.00	24.179	2.00
7.00	1.00	500.00	0.00	1.00	0.00	0.00	Inactive	3038.5	762.25	27.25	0.00	4.542	1.00
6.00	1.00	420.00	32.86	3.00	920.00	131.43	Active	1545.75	893	183.25	1.00	36.65	3.00
6.00	1.00	420.00	4.29	1.00	120.00	17.14	Inactive	1748.75	1506	202.25	1.00	28.893	3.00
7.00	0.00	120.00	17.14	2.00	480.00	68.57	Inactive	1829	849	203	1.00	29	3.00
5.00	0.00	300.00	14.29	2.00	400.00	57.14	Inactive	3582.75	2182	140.25	0.00	20.036	2.00
5.00	2.00	300.00	34.29	3.00	960.00	137.14	Active	2471.25	1132.5	150.25	1.00	21.464	2.00
5.00	2.00	90.00	8.57	1.00	240.00	34.29	Inactive	4013.75	1082.75	120.5	0.00	17.214	1.00
6.00	2.00		25.71	3.00	720.00	102.86	Active	3596	1891.75	236.25	1.00	33.75	3.00
5.00	2.00	360.00	8.57	1.00	240.00	34.29	Inactive	2574.75	920.75	109.5	0.00	15.643	1.00
5.00	1.00	420.00	2.86	1.00	80.00	11.43	Inactive	2171.25	1452.75	628	1.00	104.667	4.00
3.00	0.00		15.00	2.00	420.00	60.00	Inactive						
6.00	1.00	120.00	5.71	1.00	160.00	22.86	Inactive	4321.75	1475.75	182.5	1.00	26.071	2.00
5.00	1.00	600.00	42.86	4.00	1200.00	171.43	Active	3204.5	1373	265.5	1.00	37.929	4.00
6.00	1.00	60.00	8.57	1.00	240.00	34.29	Inactive	1708.5	1239.5	173	1.00	24.714	2.00
4.00	0.00	60.00	192.86	4.00	5400.00	771.43	Active	3099	1903.75	313.25	1.00	44.75	4.00
7.00	2.00	420.00	4.29	1.00	120.00	17.14	Inactive	3852.5	1820.25	205.25	1.00	29.321	3.00
5.00	1.00	240.00	1.43	1.00	40.00	5.71	Inactive	3604.25	1172.75	154	1.00	22	2.00
7.00	3.00	300.00	21.43	3.00	600.00	85.71	Active	2594.75	1174.75	164.5	1.00	32.9	3.00
7.00	1.00	300.00	7.14	1.00	200.00	28.57	Inactive	3199.25	892.75	148	0.00	21.143	2.00
6.00	3.00	30.00	20.00	2.00	560.00	80.00	Inactive	4397.5	1503	95.5	0.00	13.643	1.00
6.00	1.00	60.00	471.43	4.00	24720.00	3531.43	Active	3113.25	2242.25	460.5	1.00	65.786	4.00
6.00	0.00	120.00	12.86	2.00	360.00	51.43	Inactive	3231.75	902.5	275.75	1.00	39.393	4.00
6.00	2.00	180.00	85.71	4.00	2400.00	342.86	Active	2928.75	1883.25	359	1.00	51.286	4.00
5.00	0.00		6.43	1.00	180.00	25.71	Inactive	3215	1038.5	106.5	0.00	15.214	1.00
4.00	1.00	90.00	514.29	4.00	14400.00	2057.14	Active	2854.25	1386.5	208.25	1.00	41.65	4.00
7.00		660.00	20.00	2.00	560.00	80.00	Inactive	2105.5	830	167.5	1.00	23.929	2.00

6.00	2.00	[redacted]	120.00	180.00	4.00	5040.00	720.00	Active	2662.75	1031.75	257.5	1.00	36.786	3.00
6.00	1.00	[redacted]	30.00	165.00	4.00	4620.00	660.00	Active	2514.25	651.25	52.5	0.00	8.75	1.00
4.00	0.00	[redacted]	120.00	90.00	4.00	2520.00	360.00	Active	2573.5	866	74.5	0.00	10.643	1.00
4.00	0.00	[redacted]	300.00	30.00	3.00	3720.00	531.43	Active	4745	990.5	179.5	1.00	22.438	2.00
5.00	1.00	[redacted]	300.00	85.71	4.00	2400.00	342.86	Active	3144.75	1591	146.25	0.00	18.281	2.00
5.00	0.00	[redacted]	600.00	64.29	4.00	1800.00	257.14	Active	3053.75	2222.25	221	1.00	31.571	3.00
6.00	0.00	[redacted]	420.00	30.00	3.00	840.00	120.00	Active	3761.5	1024.5	107	0.00	13.375	1.00
5.00	3.00	[redacted]	420.00	98.57	4.00	2760.00	394.29	Active						
6.00	0.00	[redacted]	360.00	15.00	2.00	420.00	60.00	Inactive	3874	1315.5	289.5	1.00	41.357	4.00
5.00	1.00	[redacted]	240.00	45.00	4.00	1260.00	180.00	Active	3458.25	1549.5	211.25	1.00	26.406	3.00
5.00	1.00	[redacted]	120.00	17.14	2.00	480.00	68.57	Inactive	2008	623.25	172.75	1.00	34.55	3.00
6.00	2.00	[redacted]	180.00	12.86	2.00	360.00	51.43	Inactive	2831.25	1087.75	82	0.00	11.714	1.00
4.00	2.00	[redacted]	480.00	40.00	3.00	1120.00	160.00	Active	3891.25	1092	409.75	1.00	58.536	4.00
6.00	1.00	[redacted]	600.00	25.00	3.00	700.00	100.00	Active	2619.5	1015	115.5	0.00	19.25	2.00
5.00	0.00	[redacted]	600.00	0.00	1.00	0.00	0.00	Inactive	3188.25	472.5	45.25	0.00	6.464	1.00
6.00	1.00	[redacted]	180.00	30.00	3.00	840.00	120.00	Active	2611.75	1352.5	193.75	1.00	32.292	3.00
6.00	3.00	[redacted]	480.00	68.57	4.00	1920.00	274.29	Active	2226.75	1073.75	188.5	1.00	37.7	4.00
7.00	1.00	[redacted]		25.00	3.00	700.00	100.00	Active	3991.75	1843.5	263.75	1.00	37.679	4.00
6.00	2.00	[redacted]	390.00	77.14	4.00	2160.00	308.57	Active	3249.5	2097.5	367	1.00	52.429	4.00
7.00	3.00	[redacted]	360.00	34.29	3.00	960.00	137.14	Active	3297.25	932	72.75	0.00	12.125	1.00
5.00	2.00	[redacted]	540.00	64.29	4.00	1800.00	257.14	Active	1496.5	424.5	104	0.00	20.8	2.00
6.00	1.00	[redacted]	540.00	1.43	1.00	40.00	5.71	Inactive	4020.25	1209.5	221.25	1.00	31.607	3.00
5.00	1.00	[redacted]	540.00	8.57	1.00	240.00	34.29	Inactive	3299.5	1173.75	108.75	0.00	15.536	1.00
2.00	1.00	[redacted]	600.00	25.71	3.00	720.00	102.86	Active	2426.5	1517.25	260.25	1.00	37.179	3.00
5.00	2.00	[redacted]	540.00	15.00	2.00	420.00	60.00	Inactive	3920.25	1788.75	289	1.00	41.286	4.00
6.00	1.00	[redacted]	420.00	10.71	2.00	300.00	42.86	Inactive	2537.5	1542.75	176.75	1.00	25.25	2.00
6.00	0.00	[redacted]	360.00	2.86	1.00	80.00	11.43	Inactive	5385.5	655.75	24.75	0.00	3.536	1.00
4.00	2.00	[redacted]	720.00	4.29	1.00	120.00	17.14	Inactive	3973	1610.75	220.25	1.00	31.464	3.00
5.00	1.00	[redacted]	360.00	120.00	4.00	3360.00	480.00	Active	3222.75	1623.25	554	1.00	79.143	4.00
5.00	2.00	[redacted]	240.00	14.29	2.00	400.00	57.14	Inactive	3675.75	1419.75	248.5	1.00	35.5	3.00
5.00	0.00	[redacted]	360.00	15.00	2.00	420.00	60.00	Inactive	2272.5	577.75	73.75	0.00	14.75	1.00
7.00	0.00	[redacted]	540.00	0.00	1.00	0.00	0.00	Inactive	1654.75	517.5	43.75	0.00	7.292	1.00
6.00	2.00	[redacted]		32.14	3.00	900.00	128.57	Active	2523.75	884.5	198.75	1.00	28.393	3.00
7.00	0.00	[redacted]	120.00	51.43	4.00	1440.00	205.71	Active	3954	523	82	0.00	11.714	1.00
4.00	3.00	[redacted]	20.00	6.43	1.00	180.00	25.71	Inactive	4244.75	2184.5	608.75	1.00	76.094	4.00
4.00	1.00	[redacted]	590.00	25.71	3.00	720.00	102.86	Active	2791.25	776	142.75	0.00	20.393	2.00
5.00	1.00	[redacted]	300.00	4.29	1.00	120.00	17.14	Inactive	3639.75	1557	177.25	1.00	22.156	2.00
5.00	1.00	[redacted]	300.00	4.29	1.00	120.00	17.14	Inactive	4393.5	1166.5	291	1.00	36.375	3.00
6.00	0.00	[redacted]	150.00	94.29	4.00	3840.00	548.57	Active	3619.5	1218.25	286.25	1.00	35.781	3.00
3.00	1.00	[redacted]	300.00	30.00	3.00	840.00	120.00	Active	3466.75	1180.75	119.5	0.00	14.938	1.00
6.00	0.00	[redacted]	600.00	17.14	2.00	480.00	68.57	Inactive	3539.25	887.25	237.5	1.00	33.929	3.00
3.00	0.00	[redacted]	390.00	60.00	4.00	1680.00	240.00	Active	3723.5	1679.5	188	1.00	26.857	3.00

5.00	0.00	[redacted]	60.00	15.00	2.00	420.00	60.00	Inactive	3261	1693.25	161.75	1.00	23.107	2.00
5.00	0.00	[redacted]	120.00	77.14	4.00	2160.00	308.57	Active	3268.5	1223.75	385.75	1.00	55.107	4.00
6.00	0.00	[redacted]	240.00	12.86	2.00	360.00	51.43	Inactive	2770.5	438.25	85.25	0.00	12.179	1.00
6.00	1.00	[redacted]	450.00	68.57	4.00	1920.00	274.29	Active	1813	744	194	1.00	38.8	4.00
7.00	1.00	[redacted]	480.00	21.43	3.00	600.00	85.71	Active	3084.25	1185.25	342.5	1.00	48.929	4.00
5.00	0.00	[redacted]	240.00	21.43	3.00	600.00	85.71	Active	3445.25	1275.5	625.25	1.00	89.321	4.00
7.00	0.00	[redacted]	600.00	17.14	2.00	480.00	68.57	Inactive	3662.75	1483.75	216.5	1.00	30.929	3.00
5.00	0.00	[redacted]	120.00	308.57	4.00	16320.00	2331.43	Active	2136.75	857.5	150.75	1.00	21.536	2.00
6.00	1.00	[redacted]	60.00	17.14	2.00	480.00	68.57	Inactive	2353.5	881.75	144.75	1.00	24.125	2.00
5.00	2.00	[redacted]	300.00	6.43	1.00	180.00	25.71	Inactive	4166.5	1490.5	78	0.00	11.143	1.00
6.00	2.00	[redacted]	120.00	15.00	2.00	420.00	60.00	Inactive	1954	1448.5	376.5	1.00	53.786	4.00
6.00	1.00	[redacted]	420.00	14.29	2.00	400.00	57.14	Inactive	1305	631	61	0.00	12.2	1.00
6.00	1.00	[redacted]	240.00	21.43	3.00	600.00	85.71	Active	3877.75	1058.25	86	0.00	12.286	1.00
5.00	2.00	[redacted]	75.00	53.57	4.00	1500.00	214.29	Active						
2.00	1.00	[redacted]	300.00	17.14	2.00	480.00	68.57	Inactive	2546.5	767.75	84.75	0.00	12.107	1.00

Accelerometer_	Event Name	V5_P16_Total	V5_TotalMIN/	V5_TotalMIN/	Quartiles_V5_	V5_Ptotal_ME	V5_Ptotalday_	V5_GPAQ Mee	Sedentary	Light	Moderate	Vigorous	Very Vigorous
329.0625	**Visit 5 (29 - 33**	60.00	30.00	4.29	1.00	120.00	17.14	Inactive	1994.25	664.25	129	0.5	0
542.3571429	**Visit 5 (29 - 33**	360.00	210.00	30.00	3.00	840.00	120.00	Active	2702.25	1438	167.5	0.25	0
350.71875	**Visit 5 (29 - 33**	120.00	630.00	90.00	4.00	2520.00	360.00	Active	2627.75	679.25	64.75	0	0.25
546.53125	**Visit 5 (29 - 33**	120.00	150.00	21.43	3.00	600.00	85.71	Active	4355.25	1236.25	153	0.25	0.25
666.6785714	**Visit 5 (29 - 33**	480.00	280.00	40.00	3.00	1120.00	160.00	Active	3143.5	1115.75	152.25	1.5	0
480.3214286	**Visit 5 (29 - 33**	360.00	150.00	21.43	3.00	600.00	85.71	Active	2056.75	748.5	90.75	0	0
372.09375	**Visit 5 (29 - 33**	120.00	510.00	72.86	4.00	2040.00	291.43	Active	1556.25	379	91.5	0.25	0
452.1875	**Visit 5 (29 - 33**	540.00	50.00	7.14	2.00	200.00	28.57	Inactive					
592.0357143	**Visit 5 (29 - 33**	600.00	50.00	7.14	2.00	200.00	28.57	Inactive	2601	1358.75	254	1.25	0
399.25	**Visit 5 (29 - 33**	660.00	60.00	8.57	2.00	240.00	34.29	Inactive	879.25	238	30	1.5	0.25
678.5357143	**Visit 5 (29 - 33**	120.00	390.00	55.71	4.00	1560.00	222.86	Active	1011.25	180.5	24.5	0.75	0
551.4642857	**Visit 5 (29 - 33**	480.00	300.00	42.86	4.00	1200.00	171.43	Active	4108	1066.5	70.5	1	0
506.4166667	**Visit 5 (29 - 33**	360.00	0.00	0.00	1.00	0.00	0.00	Inactive	833.5	166.25	82.5	0.75	0
309.15	**Visit 5 (29 - 33**	300.00	280.00	40.00	3.00	1120.00	160.00	Active	2904	1060.75	167.5	1.75	0
249.8214286	**Visit 5 (29 - 33**	360.00	2000.00	285.71	4.00	8000.00	1142.86	Active	2310.25	2214.75	186.25	0.75	0
261.2857143	**Visit 5 (29 - 33**	300.00	60.00	8.57	2.00	240.00	34.29	Inactive	3189.25	722.5	73.5	2.5	1.25
511.8214286	**Visit 5 (29 - 33**	120.00	120.00	17.14	2.00	480.00	68.57	Inactive	3017.75	2468.75	128	0.5	0
353.0357143	**Visit 5 (29 - 33**	300.00	280.00	40.00	3.00	1120.00	160.00	Active	1960.5	549	41	1.25	0.25
573.3928571	**Visit 5 (29 - 33**	300.00	240.00	34.29	3.00	960.00	137.14	Active	3152	1325	57	0	0
513.7142857	**Visit 5 (29 - 33**	360.00	1200.00	171.43	4.00	4800.00	685.71	Active	2815.5	1617.25	87.75	0.5	0
367.8214286	**Visit 5 (29 - 33**	480.00	360.00	51.43	4.00	1440.00	205.71	Active	2233	950.75	183.25	1	0
361.875	**Visit 5 (29 - 33**	480.00	150.00	21.43	3.00	600.00	85.71	Active	2307.25	609.75	128.75	0.25	0
	**Visit 5 (29 - 33**	180.00	140.00	20.00	2.00	560.00	80.00	Inactive	3637	1479.75	197.75	0.5	0
617.3928571	**Visit 5 (29 - 33**	480.00	100.00	14.29	2.00	400.00	57.14	Inactive	4282	1465	378.5	0.5	0
457.7857143	**Visit 5 (29 - 33**	300.00	50.00	7.14	2.00	200.00	28.57	Inactive	2533.25	1346.5	193	2.25	0
244.0714286	**Visit 5 (29 - 33 weeks)**												
442.7142857	**Visit 5 (29 - 33**	240.00	50.00	7.14	2.00	200.00	28.57	Inactive	2680.25	1631.5	215	0.25	0
550.3571429	**Visit 5 (29 - 33**	540.00	30.00	4.29	1.00	120.00	17.14	Inactive					
514.8928571	**Visit 5 (29 - 33**	540.00	240.00	34.29	3.00	960.00	137.14	Active	2730.75	960.25	109.75	0.25	0
518.95	**Visit 5 (29 - 33**	360.00	250.00	35.71	3.00	1000.00	142.86	Active	7248.75	1959	219.75	1.5	0
457.0357143	**Visit 5 (29 - 33**	540.00	100.00	14.29	2.00	400.00	57.14	Inactive	991.25	147	14.5	0.25	0
628.2142857	**Visit 5 (29 - 33**	300.00		0.00	1.00	0.00	0.00	Inactive	3593.75	1075	90.75	0.5	0
444.75	**Visit 5 (29 - 33**	420.00	150.00	21.43	3.00	600.00	85.71	Active	5459.75	1078.25	43	0	0
461.6785714	**Visit 5 (29 - 33**	60.00	30.00	4.29	1.00	120.00	17.14	Inactive	3834.25	780	292	0.75	0
418.3928571	**Visit 5 (29 - 33**	120.00	245.00	35.00	3.00	980.00	140.00	Active	2948.75	955.75	65	0.5	0
459.2857143	**Visit 5 (29 - 33**	540.00	120.00	17.14	2.00	480.00	68.57	Inactive	3039.75	1489.25	207.75	1.25	0
570.85	**Visit 5 (29 - 33 weeks)**		105.00	15.00	2.00	420.00	60.00	Inactive	3439	1551	283.5	2.5	0
300.7857143	**Visit 5 (29 - 33 weeks)**												

380.3928571	**Visit 5 (29 - 33**	60.00	210.00	30.00	3.00	840.00	120.00	Active	999	241.25	21.75	0	0
419.0416667	**Visit 5 (29 - 33 weeks)**		50.00	7.14	2.00	200.00	28.57	Inactive					
367.6428571	**Visit 5 (29 - 33**	300.00	360.00	51.43	4.00	1440.00	205.71	Active	2481.5	1353.25	82.25	0.5	0.5
593.125	**Visit 5 (29 - 33 weeks)**												
393.09375	**Visit 5 (29 - 33**	480.00	120.00	17.14	2.00	480.00	68.57	Inactive	1901	502.25	80	10.5	0.25
436.25	**Visit 5 (29 - 33 weeks)**												
470.1875	**Visit 5 (29 - 33**	480.00	570.00	81.43	4.00	2280.00	325.71	Active	3506.75	510.75	27.75	0.75	0
	**Visit 5 (29 - 33**	120.00	780.00	111.43	4.00	3120.00	445.71	Active	6235.25	1734.25	138	3.25	0.25
553.4285714	**Visit 5 (29 - 33**	240.00	210.00	30.00	3.00	840.00	120.00	Active					
432.28125	**Visit 5 (29 - 33 weeks)**												
401.6	**Visit 5 (29 - 33**	420.00	300.00	42.86	4.00	1200.00	171.43	Active					
404.4642857	**Visit 5 (29 - 33**	360.00	90.00	12.86	2.00	360.00	51.43	Inactive	3815.5	1016.25	28	0.25	0
555.8928571	**Visit 5 (29 - 33**	360.00	480.00	68.57	4.00	1920.00	274.29	Active	4051.75	712	95.75	0.5	0
436.5833333	**Visit 5 (29 - 33**	210.00	840.00	120.00	4.00	5040.00	720.00	Active	214.5	17.5	2	0	0
455.4642857	**Visit 5 (29 - 33**	300.00	60.00	8.57	2.00	240.00	34.29	Inactive	2775	496.5	66	0.5	0
435.2916667	**Visit 5 (29 - 33**	240.00	100.00	14.29	2.00	400.00	57.14	Inactive	2483.5	1023.75	96.5	0.25	0
445.35	**Visit 5 (29 - 33**	180.00	480.00	68.57	4.00	1920.00	274.29	Active					
570.25	**Visit 5 (29 - 33**	240.00	0.00	0.00	1.00	0.00	0.00	Inactive	1722	922.25	121.75	1.75	0.25
464.2142857	**Visit 5 (29 - 33 weeks)**												
549.5416667	**Visit 5 (29 - 33**	360.00	30.00	4.29	1.00	120.00	17.14	Inactive	5320.75	1453.75	208.5	1.5	0.25
299.3	**Visit 5 (29 - 33**	360.00	210.00	30.00	3.00	840.00	120.00	Active	3047	844.25	186.75	2	0
574.3214286	**Visit 5 (29 - 33**	420.00	30.00	4.29	1.00	120.00	17.14	Inactive	4598.25	1706.5	123.25	2	0
471.3571429	**Visit 5 (29 - 33**	60.00	540.00	77.14	4.00	2160.00	308.57	Active	3004.75	777	45.5	0.75	0
346.6428571	**Visit 5 (29 - 33**	180.00	180.00	25.71	3.00	720.00	102.86	Active	3251.25	920	63	1.75	0
560.0357143	**Visit 5 (29 - 33**	180.00	420.00	60.00	4.00	1680.00	240.00	Active	3314.5	1540	263	0.5	0
362.5	**Visit 5 (29 - 33 weeks)**												
769.3571429	**Visit 5 (29 - 33**	660.00	0.00	0.00	1.00	0.00	0.00	Inactive	4737.5	688.25	59.75	0.5	0
567.5714286	**Visit 5 (29 - 33**	600.00	270.00	38.57	3.00	1080.00	154.29	Active	3182.5	1576.25	131	1.25	0
460.3928571	**Visit 5 (29 - 33**	180.00	630.00	90.00	4.00	2520.00	360.00	Active	2999.25	1050.5	186	0.25	0
525.1071429	**Visit 5 (29 - 33 weeks)**												
454.5	**Visit 5 (29 - 33**	420.00	60.00	8.57	2.00	240.00	34.29	Inactive					
275.7916667	**Visit 5 (29 - 33**	120.00	150.00	21.43	3.00	600.00	85.71	Active	3368.75	1698.75	151.25	0.25	0
360.5357143	**Visit 5 (29 - 33**	150.00	100.00	14.29	2.00	400.00	57.14	Inactive	2452.5	830.25	147.5	3.75	0
564.8571429	**Visit 5 (29 - 33**	240.00	0.00	0.00	1.00	0.00	0.00	Inactive	3797.5	587	35.25	0.25	0
530.59375	**Visit 5 (29 - 33**	300.00	45.00	6.43	1.00	180.00	25.71	Inactive	3337.75	1606.75	195	2.25	0.25
398.75	**Visit 5 (29 - 33**	360.00	45.00	6.43	1.00	180.00	25.71	Inactive	3710	1622.5	116.25	4	0.25
454.96875	**Visit 5 (29 - 33**	20.00	10.00	1.43	1.00	40.00	5.71	Inactive					
549.1875	**Visit 5 (29 - 33**	420.00	30.00	4.29	1.00	120.00	17.14	Inactive	2997	925.5	134.5	2	0
452.4375	**Visit 5 (29 - 33**	360.00	50.00	7.14	2.00	200.00	28.57	Inactive	2898.75	1178.5	246	0.75	0
433.34375	**Visit 5 (29 - 33**	120.00	15.00	2.14	1.00	60.00	8.57	Inactive					
505.6071429	**Visit 5 (29 - 33**	750.00	100.00	14.29	2.00	400.00	57.14	Inactive	1259.5	261	43	0.5	0
531.9285714	**Visit 5 (29 - 33**	120.00	210.00	30.00	3.00	840.00	120.00	Active	3034.75	712.25	64.25	3.75	0

465.8571429	**Visit 5 (29 - 33 weeks)**												
466.9285714	**Visit 5 (29 - 33**	480.00	150.00	21.43	3.00	600.00	85.71	Active	2749.25	1323	116	1.75	0
	**Visit 5 (29 - 33**	540.00	90.00	12.86	2.00	360.00	51.43	Inactive	1692.25	245.75	104.75	9.75	0.5
362.6	**Visit 5 (29 - 33**	180.00	210.00	30.00	3.00	840.00	120.00	Active	617.5	121.5	17.75	3.25	0
440.6071429	**Visit 5 (29 - 33**	300.00	20.00	2.86	1.00	80.00	11.43	Inactive	2187.25	575.75	203.5	1.25	0.25
492.1785714	**Visit 5 (29 - 33**	300.00	630.00	90.00	4.00	2520.00	360.00	Active	3478.75	946.25	438.75	1.25	0
523.25	**Visit 5 (29 - 33**	360.00	300.00	42.86	4.00	1200.00	171.43	Active					
305.25	**Visit 5 (29 - 33**	480.00	90.00	12.86	2.00	360.00	51.43	Inactive	3098.5	1786.5	115.5	1.5	0
392.25	**Visit 5 (29 - 33**	720.00	45.00	6.43	1.00	180.00	25.71	Inactive	1017.5	162	20.25	1.25	0
595.2142857	**Visit 5 (29 - 33**	300.00	120.00	17.14	2.00	480.00	68.57	Inactive	3829.75	1156.5	184.25	0.5	0
279.1428571	**Visit 5 (29 - 33**	240.00	90.00	12.86	2.00	360.00	51.43	Inactive	2415.75	902.5	134	0.75	0
261	**Visit 5 (29 - 33**	240.00	300.00	42.86	4.00	1200.00	171.43	Active	2268	636.25	101.25	0.5	0
553.9642857	**Visit 5 (29 - 33**	60.00	1200.00	171.43	4.00	4800.00	685.71	Active					
	**Visit 5 (29 - 33**	40.00	180.00	25.71	3.00	720.00	102.86	Active	3023.75	575.5	109.75	1	0
363.7857143	**Visit 5 (29 - 33 weeks)**												

Total MVPA	V5_Average M	V5_Quartile of	V5_SedentaryM	V5_Accelerom	Accelerometer	GPAQ_Change in PA
129.5	21.583	3.00	332.375	1	-12.86	-34.29
167.75	23.964	4.00	386.0357143	1	-1.29	19.29
65	9.286	1.00	375.3928571	0	-13.65	64.29
153.5	21.929	3.00	622.1785714	1	-23.04	8.57
153.75	21.964	3.00	449.0714286	1	-20.57	14.29
90.75	12.964	2.00	293.8214286	0	-54.75	10.71
91.75	15.292	2.00	259.375	0	-20.77	65.71
255.25	36.464	4.00	371.5714286	1	13.11	-21.43
31.75	5.292	1.00	146.5416667	0	-14.86	-60.00
25.25	4.208	1.00	168.5416667	0	-15.86	28.57
71.5	10.214	2.00	586.8571429	0	-13.97	0.00
83.25	13.875	2.00	138.9166667	0	9.33	0.00
169.25	24.179	4.00	414.8571429	1	-12.47	7.14
187	23.375	3.00	288.78125	1	-5.52	281.43
77.25	11.036	2.00	455.6071429	0	-17.96	-8.57
128.5	18.357	3.00	431.1071429	0	-1.68	2.86
42.5	6.071	1.00	280.0714286	0	-15.39	5.71
57	8.143	1.00	450.2857143	0	-9.07	25.71
88.25	14.708	2.00	469.25	0	-19.04	145.71
184.25	26.321	4.00	319	1	10.68	42.86
129	21.5	3.00	384.5416667	1	-83.17	18.57
198.25	28.321	4.00	519.5714286	1	28.32	5.00
379	54.143	4.00	611.7142857	1	28.07	8.57
195.25	27.893	4.00	361.8928571	1	-10.04	-35.71
215.25	35.875	4.00	446.7083333	1	-8.88	-185.71
110	18.333	3.00	455.125	0	-3.67	32.86
221.25	31.607	4.00	1035.535714	1	-1.29	14.29
14.75	2.107	1.00	141.6071429	0	-19.04	7.14
91.25	13.036	2.00	513.3928571	0	-0.61	-20.00
43	6.143	1.00	779.9642857	0	-59.64	-450.00
292.75	36.594	4.00	479.28125	1	-2.80	-8.57
65.5	10.917	2.00	491.4583333	0	-40.37	-50.71
209	29.857	4.00	434.25	1	14.64	10.71
286	40.857	4.00	491.2857143	1	-0.79	-499.29

21.75	4.35	1.00	199.8	0	-32.44	-150.00
83.25	13.875	2.00	413.5833333	0	3.23	-38.57
90.75	12.964	2.00	271.5714286	0	-5.32	-68.57
28.5	4.071	1.00	500.9642857	0	-9.30	51.43
141.5	20.214	3.00	890.75	0	20.21	12.86
28.25	4.036	1.00	545.0714286	0	-7.68	0.00
96.25	13.75	2.00	578.8214286	0	-44.79	28.57
2	0.5	1.00	53.625	0	-18.75	95.00
66.5	9.5	1.00	396.4285714	0	3.04	8.57
96.75	12.094	2.00	310.4375	0	-20.20	-15.71
123.75	20.625	3.00	287	0	-17.05	-25.00
210.25	10.513	2.00	266.0375	0	-1.61	-30.00
188.75	23.594	4.00	380.875	1	2.79	-34.29
125.25	13.917	2.00	510.9166667	0	-17.69	2.86
46.25	7.708	1.00	500.7916667	0	-7.83	68.57
64.75	9.25	1.00	464.4642857	0	-27.93	0.00
263.5	43.917	4.00	552.4166667	1	2.63	45.00
60.25	8.607	1.00	676.7857143	0	5.07	-2.86
132.25	18.893	3.00	454.6428571	0	-12.57	34.29
186.25	31.042	4.00	499.875	1	-48.10	-30.00
151.5	21.643	3.00	481.25	1	14.35	21.43
151.25	21.607	3.00	350.3571429	1	-6.79	-17.86
35.5	5.071	1.00	542.5	0	-6.64	-51.43
197.5	19.75	3.00	333.775	0	-56.34	0.00
120.5	17.214	3.00	530	0	-3.18	-19.29
136.5	17.063	3.00	374.625	0	-19.31	0.00
246.75	30.844	4.00	362.34375	1	-4.94	-87.14
43.5	10.875	2.00	314.875	0	-23.05	-2.86
68	9.714	2.00	433.5357143	0	-17.14	-30.00

117.75	19.625	3.00	458.2083333	0	-35.48	-55.71
115	16.429	2.00	241.75	0	4.25	0.00
21	4.2	1.00	123.5	0	-34.60	-38.57
205	29.286	4.00	312.4642857	1	-19.64	-18.57
440	62.857	4.00	496.9642857	1	-26.46	68.57
117	16.714	3.00	442.6428571	0	-4.82	-295.71
21.5	3.583	1.00	169.5833333	0	-20.54	-10.71
184.75	26.393	4.00	547.1071429	1	15.25	10.71
134.75	16.844	3.00	301.96875	0	-36.94	-2.14
101.75	14.536	2.00	324	0	2.34	28.57
110.75	15.821	2.00	431.9642857	0	15.82	-27.86
